# Peer-Assisted Learning for First-Year Nursing Student Success and Retention: Findings from a Regional Australian Study

**DOI:** 10.3390/nursrep15070252

**Published:** 2025-07-10

**Authors:** Andrew Woods, Fiona Lotherington, Paula Steffensen, Theane Theophilos

**Affiliations:** Faculty of Health, Southern Cross University, Lismore, NSW 2480, Australia; fiona.lotherington@scu.edu.au (F.L.); paula.steffensen@scu.edu.au (P.S.); theano.ratcliff@sim.org (T.T.)

**Keywords:** peer-assisted learning, clinical laboratory, retention, undergraduate students, nursing, inclusive pedagogy, academic performance

## Abstract

**Background/Objectives**: In Australia, attrition rates in undergraduate nursing degrees have been increasing nationally. The aim of this study was to explore if and how clinical laboratory-based peer-assisted learning (PAL) improved the first-year nursing student learning experience and retention at a regional university. A further aim was to explore any perceived benefits for third-year student participants. **Methods**: This is a descriptive study design. The study recruited eight third-year nursing students (named ‘LabPALs’) offering support to 42 first-year nursing students during their self-directed laboratory practice sessions. The first-year students included a high percentage of mature aged and ‘first in family’ students. Over an eight-week period, LabPALs provided peer support for up to four students per one-hour practice session. Unit grade outcomes were compared with students not exposed to the PAL sessions. Both the LabPAL mentors and first-year participants were asked to evaluate their experience. **Results**: It was found that PAL project participation was associated with higher completion rates when compared with non-participation. When combined with exposure to their experienced peers’ perspectives and support, participation was associated with academic success. Thematic analysis found that first-year nursing students reported developing both ‘confidence’ and ‘competence’ in their laboratory learning spaces. The third-year LabPAL students reported skills gained in facilitating peer learning and perceived their experience as very rewarding. **Conclusions**: This research suggests that PAL enhances clinical laboratory learning among undergraduate nursing students.

## 1. Introduction

This paper reports the findings of a pilot program that focused on peer-assisted learning (PAL) for undergraduate nursing students in clinical laboratory settings. The study involved third-year students providing support to first-year students to prepare for clinical assessments at an Australian regional university. Underpinned by a constructivist theoretical framework based on Vygotsky’s zone of proximal development [1], the study aligned with the ‘near-peer’ model of PAL, where student learning is enhanced through social interaction with more experienced peers from a similar education program [2].

Modelled on the successful Peer-Assisted Study Sessions (PASS) program [3], the program was designed in response to undesired Bachelor of Nursing (BN) first-year student nurse attrition rates. With a focus on first-year student retention, the goal was to improve the learning experience and success of first-year students. In alignment with United Nations sustainable development goal number 4 [4], a secondary goal was to provide equitable and inclusive education for students with backgrounds that present increased academic risk. Tailored support for early higher education academic success has been linked to lifelong learning skills [5], wellbeing and retention [6].

In addition, the program provided third-year nursing students with a professional development opportunity. The PAL role aligned with national standards for nursing practice which specify the requirement that nurses facilitate the clinical development of their peers [7]. These requirements also feature in section five of the Australian National Code of Conduct for nurses, which states “nurses commit to teaching, supervising and assessing students and other nurses in order to develop the nursing workforce across all contexts of practice” [8] (p. 13).

### 1.1. Background

In Australia, the gap between Bachelor of Nursing (BN) student commencements compared with completions has been growing. This gap was recognised in a Health Workforce Australia (HWA) Nurses in Focus report [9], which resulted in government stimulus for further BN enrolments, rather than targeted student engagement and retention [10].

In the context of a HWA projected shortage of over 100,000 nurses by 2025, the retention of nursing students is vital to sustain a viable healthcare system [11]. Initiatives to retain students in nursing programs are guided by identified factors that contribute to attrition and the impacts on higher education institutions as well as the sustainability and quality standards of the profession [12]. Internationally, attrition rates from BN courses in Europe (10–33%), the United States (20%), Australia, and Canada (10–50%) are an ongoing challenge [13]. In the period prior to the COVID-19 pandemic, a systematic review of student nurse attrition identified academic failure and clinical performance as the main risk factors for attrition [14]. A survey of Australian nursing students’ support needs during the pandemic identified social isolation and the need for additional learning support [15]. Since the COVID-19 pandemic, multiple further risk factors, such as the cost-of-living crisis, have impacted the university experience. In some regional areas of Australia, further stressors have included fire and flood events. In a systematic review of factors that influence nursing student retention in regional and remote Australia, the authors found limited evidence of how extra-academic support programs were effective [16].

In nursing programs, attrition has also been associated with vulnerable student groups, namely mature-aged students, ‘first in family’ tertiary students (defined as students who are the first to attend university in their immediate families), students with low socioeconomic status (SES) and underrepresented cultural backgrounds [12]. Mature-aged students are defined here as any student who is a not a current school leaver. A survey of mature-aged nursing students (*n* = 121) returning to Australian regional universities found that this cohort were time-poor, lacked self-confidence and would choose to persist with their degree if they experienced stronger connection to their learning journeys [17]. These students typically have family and employment responsibilities that reduce time on campus and learning engagement [18].

At the targeted regional university, student retention data demonstrated that BN course attrition rates had increased from 22.6% in 2011 to 32.2% in 2016, figures that matched the national trend. In response, the university introduced a ‘student engagement and retention team’ as well as programs for academic peer support including PASS and a student mentor program. Internationally, student participation in peer learning has been linked to higher course grades and retention [19]. In New Zealand, institutional support factors, including peer mentoring, has been identified as a factor in Māori student nurse retention and success [20]. Leading up to the time of the study, BN student feedback consistently requested additional support during laboratory practice times, thus presenting an opportunity to introduce clinical laboratory peer support.

In the undergraduate healthcare context, PAL studies have reported better clinical or skill performance outcomes when compared with those reporting on academic success. In medical education, a systematic review found that PAL was best suited to the learning of practical and procedural skills associated with improvement in practical assessment outcomes [21]. In nursing education, a scoping literature review focused on the impact of PAL found more evidence of improved practical (skill-based) outcomes compared with theoretical/academic outcomes [22]. In nursing contexts, third-year mentorship for first-year students has been found to improve clinical laboratory skill-based performance outcomes [23]. Near-peer student support during simulation learning and clinical skill acquisition was found to lead to increased self-efficacy and confidence when performing tasks, compared with the non-PAL control groups [24,25]. The formation of professional identity, socialisation and soft skill enhancement has been found in nursing clinical placement contexts [26]. This literature suggests that the clinical laboratory environment has potential to extend the benefits of university academic PAL and mentor programs.

BN clinical laboratory-based units offer unique learning experiences, with students integrating theoretical knowledge with practical skills in preparation for clinical placement experiences. The laboratory environment and equipment offer students opportunities for kinaesthetic and visual learning as they engage in authentic simulated clinical contexts. An Australian study found that first-year nursing students from rural/regional contexts had significantly higher visual and kinaesthetic learning preference scores compared with metropolitan counterparts [27]. Success for students with these learning styles is related to gaining confidence through practicing clinical skills (for example, accurately measuring blood pressure) for clinical skill viva voce-style assessments. In addition, the laboratory space, by authentically simulating ‘real world’ clinical environments, provides opportunities for socialisation to professional roles and identities [28,29]. Success in laboratory contexts extends academic success, as students connect to nursing through peer learning communities [30]. This early socialisation relates to the early formation of professional identity, a sense of belonging and future direction [2,31]. Whilst the introduction of more experienced third-year peers to the clinical laboratory environment may extend these learnings, caution is required to ensure clear role delineation and expectations.

With additional training, third-year students who have experienced years of clinical laboratory learning are well placed to take up near-peer PAL roles. However, the PAL literature has reported that students can be under-prepared for such roles. A review of near-peer teaching in undergraduate nursing education found a lack of curriculum-based training for near-peer mentors and tutors [32]. Several studies have reported specifically on third-year nursing students involved in PAL for first or second-year students [31,33,34,35]. These studies have reported benefits for third-year students, including “more confidence” and being “better prepared for practice;” however, they also found limited positive learning outcomes for first-year students. In all of these studies the third-year students undertook tutor-style roles, including co-teaching with their tutor. This was found to be problematic as the third-year students reported being “inadequately prepared” and lacking teaching knowledge [35].

An alternative to scheduled laboratory classes involves the introduction of PAL to laboratory practice sessions, where learning is less structured and student driven. Qualitative findings in the literature highlight that student peers communicate with language that first-year students are more likely to understand and feel comfortable with [21,22]. Near-peer students are also likely to remember psychosocial factors that contribute to clinical laboratory learning being an unfamiliar experience for first-year students [28]. With support from more experienced third-year students, first-year students can clarify what to expect and how to practice and better prepare for assessment tasks, helping them adapt more successfully to these learning contexts [36]. For third-year student facilitators, confidence gained from their experience can contribute to successful completion of their course, transition to practice and improve their employability through evidence of leadership, educational and peer support skills for their professional portfolio [37,38].

### 1.2. Study Aim

The aim of this mixed-methods study was to explore if and how clinical laboratory-based peer-assisted learning (PAL) improved first-year nursing student learning success and retention at an Australian regional university. A secondary aim was to explore any benefits for third-year participants who acted as LabPALs. First-year students were asked if and how the PAL sessions benefited their learning experience and intention to continue their studies. Third-year students were asked if and how they benefited from their PAL experience.

## 2. Materials and Methods

A social constructivist lens provided the theoretical framework for the PAL program. Social constructivism advocates that successful teaching and learning is contingent on interpersonal interactions and discussion, with the focus on understanding the discussion [39]. In particular, Vygotsky’s theory of the zone of proximal development outlined the social origins of understanding and development where people with common or shared interest and language benefit from exposure to more experienced peers [1]. Extending traditional student–teacher models of learning, PAL promotes social proximity and phenomenological congruence of student peers, who, through shared understanding of learning challenges, can relate better to their peers than academic staff [2].

The study used a descriptive research design. For data generation, both quantitative and qualitative methods were utilised with an emphasis on qualitative analysis. The quantitative analysis focused specifically on unit grade outcomes triangulated to student perceptions of success [40]. Following ethics approval (ECN-16-050) from the University Human Research Ethics Committee, recruitment messages were added to first and third-year online learning sites. Drawing on programmatic methods employed by Goldsmith et al. [23] and Pegram and Fordham-Clarke [36], three one-hour practice sessions were scheduled for one day of the week over an eight-week period (24 sessions in total) with a ratio of one LabPAL facilitating up to four first-year students.

In 2017, based on convenience sampling, all first-year students enrolled in a BN laboratory-based learning unit, *Skills Platform for Nursing Practice,* at the intervention campus (*n* = 88) were invited to participate in the program. The researchers were not involved in the teaching, assessments or grading in this unit. An instruction portal was added to their unit site with information about booking sessions and LabPAL availability. Being a self-section supplementary learning activity, all students had the opportunity to book practice time. Any students booking into a practice session were sent an invitation and their consent was sought to participate in the research by completing a survey. A participant information sheet stated that participation was voluntary and that choosing to participate or not would have no impact on the availability of practice time. Confidentiality was assured as all data were deidentified and no records of participation were to be shared with the teaching team. Students were included in the study if they participated in one or more facilitated clinical laboratory practice session with a LabPAL. Students who did not book a practice session, or who chose not to book a LabPAL were not included in the study survey.

Third-year students (*n* = 50) enrolled in a BN capstone unit, *Care Management and Leadership*, (at the same campus) were invited to submit a written expression of interest for the LabPAL role. The research team (composed of academic, clinical supervision and laboratory-based technical staff) screened applications. Students were excluded if they had a record of professional misconduct or academic conduct issues. Successful applicants (*n* = 8) consented to participate in the pilot program and research on a volunteer basis. A one-day face-to-face orientation session was provided for the LabPALs. Participants additionally consented to participate in filmed interviews to help inform and orientate future LabPALs. The generation of these videos and other resources contributed to the development of an online *Blackboard* orientation site set up to facilitate LabPAL training and the sustainability of the program. The LabPALs were asked to be available for at least two practice sessions (two hours minimum) with the aim of covering all 24 practice sessions between them (average of three hours per LabPAL over the eight weeks) and were not paid.

Following the final PAL session, surveys were circulated to all participants. Open-ended questions were utilised to elicit qualitative data for thematic analysis [41]. The first-year students were asked to write about their experience, whether and how they thought the LabPAL sessions were beneficial to their learning, and how the sessions related to their confidence and engagement with unit assessments tasks. In addition, they were asked if their experience had contributed to their decision to continue their nursing studies. Third-year students were asked to write about their LabPAL experience and how they perceived this to benefit their own learning and professional development. In accordance with Braun and Clarke’s [42] six stages of thematic analysis, three members of the research team analysed the raw qualitative survey data for both the first and third-year participants. One researcher used QSR International’s NVIVO 11 (2018) and two manually coded the data. Each researcher then noted each major theme as a “node.” The nodes were then discussed in relation to coded extracts until the research team identified defined themes [41]. The resultant themes were then analysed in the context of the research questions and background literature. Using multiple perspectives can add breadth to phenomena of interest and help confirm the final themes [43]. The researcher triangulation process helped to ensure that the qualitative data analysis presented was trustworthy [44].

First-year participant unit success was compared with a quasi-control group of students enrolled in the same unit who did not participate in any PAL practice sessions. Quantitative unit grade data (*Blackboard* Grade Centre) were analysed using the International Business Machines’ (IBM) Statistical Package for Social Sciences (SPSS) version 25. A chi-squared test of association was used to analyse the proportion of the PAL-exposed students who successfully completed their laboratory unit compared with the control students. In addition, an independent *t*-test was undertaken to compare mean unit assessment scores for PAL program students and students from the control group.

## 3. Results

### 3.1. First-Year Participants

Forty-two first-year students participated in the PAL pilot with 27 completing the survey (64% response rate). Over 70% of the participating students met the traditional definition of a mature-aged student. Table 1 presents demographic data of the PAL participants. For comparison purposes, demographic data for the non-participating (control) first-year students (*n* = 46) were obtained from university records. More PAL participants reported being from a low SES background compared with non-PAL control group (30.4% vs. 26.9%) but less reported being first in family (51.8% vs. 67.3%).

Grade data collected included overall grade score and successful completion rates for students enrolled in the first-year unit. PAL project participation resulted in *n* = 41 (97.6%) students successfully completing the unit. Students not exposed to PAL support had a lower (*n* = 35, 80.4%) successful completion rate. It was found that the PAL project participation was associated with a higher completion rate compared with non-participation, χ^2^(1, 88) = 6.44, *p* = 0.012. We note, however, that there are confounding factors beyond the LabPAL intervention which may also be attributable to enhanced completion. This study was not powered to detect these.

**Figure 1:**
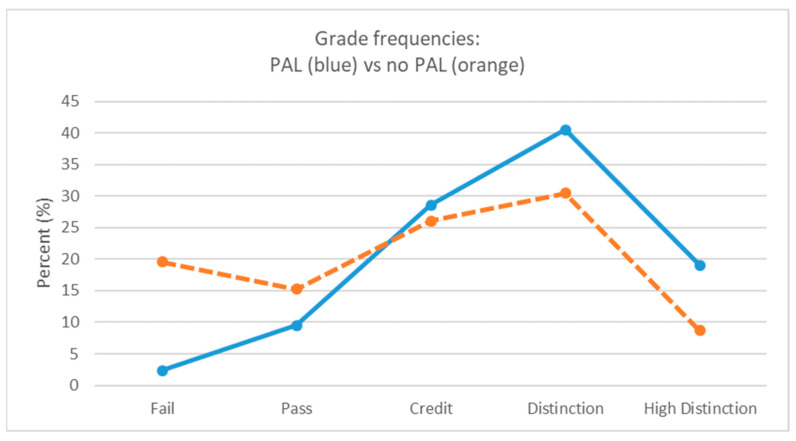
Final unit grade frequencies (fail to high distinction) with LabPAL versus no LabPAL.

First-year participants had higher unit total assessment scores (mean = 76.1, median = 77.3, SD 8.9) compared with their peers who did not participate (mean = 69.6, median = 72.4, SD 14.7). These differences were found to be statistically significant t(86) = 2.48, *p* = 0.015. For unit grade calculations, a score of 65–74 resulted in a credit and a score of 75–84 resulted in a distinction. Figure 1 presents the grade frequencies for participants exposed to a LabPAL and those not exposed to a LabPAL. The PAL project participants averaged a ‘distinction’ and non-participants a ‘credit’.

#### 3.1.1. First Year BN Attrition and Retention Data

At the study university, data provided by the university statistics department for BN student retention demonstrated a sustained improvement in continuing student retention rates. Due to COVID-19 and an associated change in the university data collection system, retention data are only available from the period 2020–2024. However, first-year students exposed to the program at the intervention campus during 2017–2019 would be captured in continuing retention data up until 2021. As multiple extenuating factors may have influenced the validity of these data, no statistical analysis is presented.

In 2020, the BN continuing retention rate at the intervention campus was 89.7%. This was higher than the two other university campuses at 82.1% and 79.9%. The combined BN continuing retention of 82.1% was also higher than the university-wide rate of 76.2%. This trend has continued to 2024, where the BN continuing rate of 91.3% remains higher than the university rate of 79.4%.

The overall BN course attrition rate of 32.2% in 2016 dropped to 21.3% in 2020. In 2020, the intervention campus attrition rate at 12.3% was lower than the other campuses. Through to 2024, attrition rates have continued to fall; commencing 16.2% and continuing 5.6%. However, the university introduced multiple other programs and curriculum changes during that time period.

#### 3.1.2. First-Year Qualitative Responses

**Figure 2:**
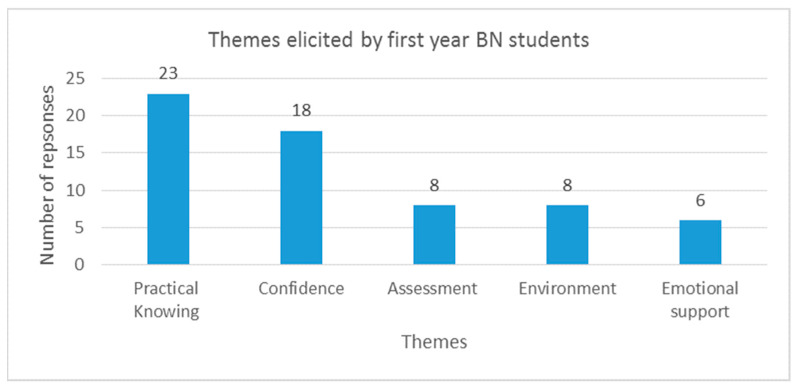
First-year student participant themes.

The majority of comments made by first-year participants related to the themes of ‘practical knowing’ and gaining confidence. Along with assessment, environment and emotional support, the number of statements relating to each theme is presented in Figure 2. Students also made a number of comments relating to the benefits of having more time with their LabPAL (compared with tutor) as well as the practice sessions contributing to their decision to continue with their studies. The themes were not mutually exclusive and many overlapped with one or more others (for example, support was linked to gaining confidence).

##### Practical Knowing

Above all, the first-year student participants expressed a sense of ‘practical knowing’ (23 related comments) associated with learning to apply theory to practice. This theme included comments about gaining feedback, knowledge of the LabPAL’s perspective and tacit knowledge expressed as helpful hints and tips. Knowledge that the LabPALs had successfully passed their skills-based assessments was expressed as encouraging in several comments. The following student comment expressed this sense of practical knowing.

*PAL sessions were absolutely beneficial to my learning. Having an opportunity to go over skills learnt in class and being guided by third-year students definitely helped me to refine skills learnt and to turn theory into practical knowledge and skills. Knowing third-year students had learnt these skills & passed the same assessments less than 2 years ago was very encouraging in the “if they can do it” sense.*

##### Confidence

Many students expressed comments associated with gaining confidence, particularly in relation to assessment tasks.

*Extremely beneficial to my learning as I could book into numerous PAL sessions and practice until I was confident.*

*Helped me feel more confident in my assessments.*

One student expressed how gaining confidence was empowering.

*Gave me confidence and knowledge that impacted my ability to get a good grade by empowering me to practice and learn more.*

A further student commented on gaining confidence from knowing others succeed under similar personal circumstances.

*Confidence in my skills that others had done it with added “life pressure” like myself and succeeded.*

##### Assessment

Eight responses suggested participation helped students prepare for clinical skill assessments.

*It allowed us to work on our scenarios, which overall helped when the assessments were on.*

Students also expressed a sense of personal development extending beyond their unit to clinical placements.

*Helped me to feel more confident in my assessments for when I go on my professional experience placement.*

##### Environment and Emotional Support

Several first-year students made statements relating to the laboratory learning environment, including a sense of belonging, emotional support and reassurance.

*Made me feel a part of the student nurse community.*

*Third-year students were supportive.*

Several comments related to reassurance gained from the LabPAL’s perspective of coping. This provided new insights related to the decision to continue in the BN course.

*Talking to third years also allowed us to see what it would be like if we continued in our studies.*

*Third-year students were encouraging & excited about the course which promoted drive to study and positivity around a career in nursing.*

*Showed me what my pathway looked like.*

One participant linked support and role-modelling to their professional socialisation.

*Third-year students I encountered were exceptional in their communication and instruction. Observing them speak & conduct themselves like qualified nurses made me realise that over the course of time the 3rd years had gained all the skills needed to communicate like a professional and complete clinical tasks with competence, even though they too struggled at the beginning. This in itself was a huge boost of encouragement and confidence.*

### 3.2. Third-Year Student LabPALs

Eight third-year students volunteered as LabPALs with 89% (*n* = 7) completing the survey. All of the LabPALs were full-time students and four were mature aged. Four of the group had previously participated in the UniMentor program and two as PASS instructors.

#### 3.2.1. LabPAL Qualitative Responses

**Figure 3:**
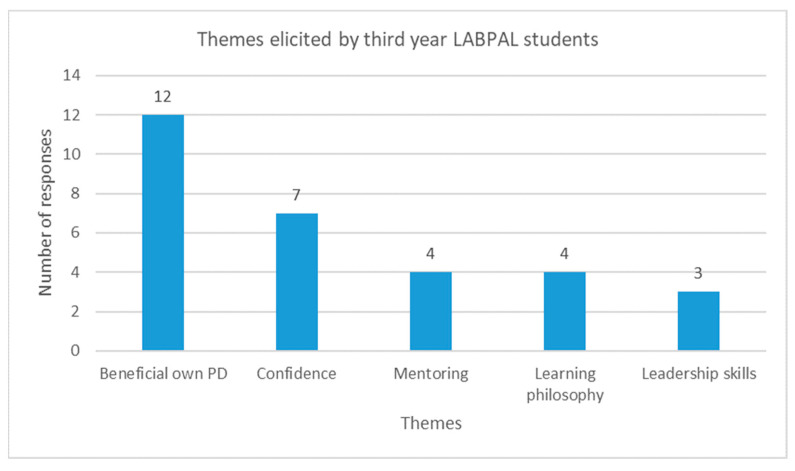
Third-year student (LabPALs) themes.

The five main themes found from the LabPAL qualitative responses are presented in Figure 3.

##### Professional Development

Above all, the seven LabPALs expressed how participation in the PAL project was beneficial to their own professional development and preparation for employment.

*I thought the project was really beneficial—it helped me cement my own knowledge and made me realise how far I’d come in terms of my own clinical knowledge and experience… It made me wish that I’d had a similar thing happening when I was a first-year student.*

*As a third-year student the program provided me with a unique opportunity to develop my skills as a clinician and provided me with a competitive edge for graduate employment.*

##### Confidence

Many of the responses expressed a sense of confidence gained that included attributes related to professional role expectations such as sharing knowledge and preceptorship.

*I now have confidence in myself that I am able to help future nurses develop their skills and knowledge.*

*I believe this experience has given me the confidence to take on a preceptor role in the future.*

One student linked their confidence gained to professional autonomy.

*This program has given me more confidence to work alone without support.*

##### Mentoring

Responses related to mentoring included both personal (behavioural) aspects as well as broader discipline awareness.

*This program has reminded me of a number of things regarding nursing and mentoring. To me this closely involves culture and having an attitude that is gentle but sure and as least intimidating as possible.*

One comment also demonstrated an awareness of not enacting the tutor role with the following metaphor.

*I found it challenging at times to remember that you are not their teacher, but more of a guide to point them in the right direction.*

##### Learning Philosophy

Several responses expressed knowledge of principles associated with adult learning. The comments were consistent with a philosophy of PAL and offered insights relevant to contemporary pedagogies and the relational aspect of learning.

*It also reinforced that as adults, we learn differently and at different rates.*

*The new students find the answers with their own reasoning.*

*I did find the role beneficial to my own learning as it highlighted that to effectively guide a learning experience, as the guide, you had to adapt a variety of communication styles that conveyed the message or experience that you were sharing with the student.*

##### Leadership Skills

The following comments captured the theme of leadership skills.

*The leadership unit that ran this session aligned very well with the project as I was able to practice leadership styles I was learning.*

*The program allowed me to enhance and develop my leadership skills in a comfortable yet professional environment.*

*Transformational leadership as a key element reminded me to be inspirational, positive and encouraging.*

Finally, a few responses highlighted some of the challenges faced by the third-year students enacting the LabPAL role.

*More than one person assisting is needed as it was difficult to give everyone attention*

*I would have preferred it to not have clashed with a fairly intense semester.*

## 4. Discussion

Whilst the benefits of academic and workplace mentoring have been recognised for undergraduate nursing students [45], PAL has been described as having an ‘untapped potential’ [46]. This study has demonstrated that, beyond student mentor and academic peer support programs, laboratory learning spaces are well-suited for targeted extensions of PAL in higher education settings. The findings of this study suggest that attending additional laboratory practice sessions supported by a LabPAL has multiple beneficial outcomes.

A key finding of this study was the sense of increased confidence expressed by both first- and third-year students. A systematic review of peer learning in undergraduate nursing education highlighted self-confidence as one of the main positive outcomes associated with multiple beneficial outcomes [47]. Confidence can be related to successful engagement which, in turn, can build more confidence through knowledge of achievement. First-year participants reported being encouraged by the third-year students’ success and insight into their BN journey, seeing their third-year peers as professional role models who had managed to complete clinical tasks with competence, even though they too struggled at the beginning. A systematic review of PAL qualitative studies involving undergraduate nursing students found that confidence and increased social and communication skills contributed to first-year student self-efficacy and resilience [48].

The study’s quantitative findings of unit assessment scores, completion rates and grades help to contextualise the qualitative findings related to assessment preparation and confidence. These results are consistent with Vygotsky’s zone of proximal development where exposure to more experienced students can enhance student learning and professional socialisation. This suggests that, in lieu of student–teacher power dynamics, the sociogenic interactions between the near-peer students resulted in what Eun [49] has referred to as the co-construction of understanding through collaborative efforts.

There is evidence that first-year student success can extend beyond initial instances of PAL experiences. A systematic review of medical student clinical skill PAL found that students participating in PAL performed significantly better than non-participants when assessed one month after teaching ended [21]. This suggests that students exposed to PAL may adopt lifelong learning skills from their peers. McKenna and Williams [2] described such phenomena as the hidden curriculum in near-peer learning. Along with a range of positive behavioural outcomes, these authors also warned of possible negative outcomes (for example, shared personal beliefs and stereotypes) that can be negotiated with carefully managed PAL encounters.

From the evidence of the current study, it is hypothesised that exposure to PAL in nursing laboratories is associated with multiple factors, that, in combination have the potential to influence first-year student participants’ decisions to continue their studies. Beyond a sense of practical knowing, the first-year themes of confidence and emotional support conveyed a sense of reassurance gained from the PAL experience. In a systematic review that explored strategies to improve student nurse retention, a sense of belonging, self-confidence and self-efficacy appeared to make students more likely to remain in their course [50]. Sheikoleslami et al. [13] highlight that strengthening supportive environments is a key strategy for improving retention amongst undergraduate nursing students. The authors of the current study assert that the PAL program was a form of institutional social and learning support that likely contributed to the university’s BN retention data.

Initiatives that involve PAL support for laboratory learning spaces may hold additional value in rural and regional settings. In Australia, higher attrition rates in these settings remain under-explored and further research will help to inform targeted interventions [51]. Rural and regional universities have higher percentages of vulnerable students, including mature-aged students with families and work commitments who are time-poor [17]. Students have had to contend with the COVID-19 pandemic and the cost-of-living crisis, which has contributed to higher rates of anxiety and depression amongst nursing students [52]. The unfamiliar clinical laboratory learning space and skill assessments (OSCE) performance expectations present additional challenges and stressors. Rather than focusing on the vulnerabilities of first-year students, the current study focused on the strengths of third-year students to provide peer support.

In the current study, the careful selection and orientation of LabPAL students helped to maximise the benefits for first-year student learning. In clinical laboratory learning environments, students can experience negative feelings in relation to performing skills or tasks in front of their class or tutor [53]. For our PAL practice sessions, tutor pre-recorded video demonstrations were available via the students’ learning sites and LabPALs were asked to avoid performing skill demonstrations themselves. This highlights the need to carefully consider the preparation of student mentors and facilitators with regard to boundaries and the need to avoid the replication of tutor-style roles. Broader curricula considerations should include student preparation to enact collegial supportive roles and mentorship.

For the third-year LabPALs, the findings build on previous research that has found that third-year students feel more confident and better prepared for mentorship roles associated with nursing practice [31]. Consistent with Dennison [46], the third-year findings in this study confirm that the PAL role is a rewarding experience. The third-year students reflected on how they had consolidated previously learnt clinical skills whilst also developing the interpersonal skills required to facilitate peer learning. These PAL skills help to prepare students for nursing practice [34]. The LabPAL program was also designed to help prepare nursing students for leadership roles [54,55]. Despite a third-year BN study load, students “stepped up” to the role and ensured all PAL practice sessions were adequately covered. These are important attributes that link PAL to the development of future nurse educators.

Whilst the university attrition and retention data are suggestive that the PAL program had a positive impact, the many variables and extenuating circumstances limit any attempt to claim an associative significant impact. The research team believe that the project was part of a wider culture of change and investment in student support projects that resulted in improved BN student retention.

### Limitations

The relatively small convenience sample was a limitation. Additionally, the one-time survey combined with potential participant self-selection bias limit the statistical findings of the study. Further exploration of the non-participant group would have deepened the analysis of quantitative comparisons. For these reasons, caution is required to generalise the quantitative findings of this study to other contexts. These limitations were partially mitigated by the inclusion of qualitative data to identify themes and factors that have been identified in the literature as being important to student decisions to continue with their studies.

For future research in similar contexts, it would be valuable to conduct a larger study across multiple (regional and metropolitan) centres. To identify a sustained impact of the PAL program, a longitudinal study could include other factors that may correlate with student attrition trends, such as changes to curricula or external events and crises.

## 5. Conclusions

The PAL program was able to harness the potential of semi-structured near-peer learning support. The first-year BN students were able to develop both confidence and competence in unfamiliar laboratory learning spaces. The study found evidence of improved completion rates and learning outcomes for first-year students. The authors believe that the context of clinical skill and assessment practice sessions and avoidance of tutor-style PAL roles enhanced the social connections and collaboration between the students. The third-year LabPALs valued the opportunity to review previous learning and gained satisfaction from supporting their peers. They also gained skills and attributes relevant to professional nursing standards and codes of conduct. The ability to help instil a sense of practical knowing and peer self-efficacy provides a valuable contribution to both BN students and the future of nursing practice.

Beyond consolidating clinical skills, the additional socialisation and reassurance may have helped to anchor first-year students to their BN learning experience. Whilst confirming PAL findings from previous similar studies, this is the first study of PAL for nursing students from a regional Australian context that included both qualitative thematic findings with descriptions of unit grades.

This study supports the view that the structured integration of PAL into nursing curricula can contribute to clinical laboratory completion rates for students with risk factors for early course attrition. Future studies that explore this relationship in other contexts should consider experimental research designs to help identify mechanisms or associative evidence of PAL on retention rates. The reversal of historically high BN attrition rates remains an important factor in meeting rural and regional workforce nursing shortages.

## Figures and Tables

**Table 1:** First-year student nurse PAL project participant demographics (n = 27).

Demographic	Category	n	%
Age (years) ^1^	17–20	6	23
	21–25	7	27
	26–35	7	27
	36–45	4	15
	>45	2	8
First in family		14	52
Low SES background ^2^		8	30
Previous PAL	PASS	3	11
	Other ^3^	5	18

^1^ One age not specified. ^2^ Self-reported. ^3^ Unimentor and non-disclosed.

## Data Availability

The raw data supporting the conclusions of this article will be made available by the authors on request.

## Public Involvement Statement

The third-year student participants were provided with an orientation workshop to the LabPAL role. This included university policies and support mechanisms available to them when interacting with the first-year student participants. For all student laboratory practice sessions, a staff member was on site to ensure student participant safety.

## Guidelines and Standards Statement

This manuscript was drafted against the STROBE (The Strengthening the Reporting of Observational Studies in Epidemiology) for observational research.

## Abbreviations

The following abbreviations are used in this manuscript:

PAL	Peer-assisted learning
LabPAL	Laboratory-based PAL
PASS	Peer-assisted study sessions
SES	Socio-economic status
